# Evaluating the Implementation of fan-beam CT-guided online adaptive re-planning in definitive cervical cancer radiotherapy

**DOI:** 10.3389/fonc.2025.1509619

**Published:** 2025-03-21

**Authors:** Shuai Sun, Xinyue Gong, Yongguang Liang, Yuliang Sun, Dan Que, Yangchun Xie, Shumeng He, Lei He, Hao Liang, Yijun Wang, Xinyi Wu, Cheng Wang, Bo Yang, Jie Qiu, Ke Hu, Fuquan Zhang

**Affiliations:** ^1^ Department of Radiation Oncology, Peking Union Medical College Hospital, Chinese Academy of Medical Sciences and Peking Union Medical College, Beijing, China; ^2^ Eight-Year Program of Clinical Medicine, Peking Union Medical College, Chinese Academy of Medical Science and Peking Union Medical College, Beijing, China; ^3^ Department of Oncology, The Third Affiliated Hospital of Chongqing Medical University, Chongqing, China; ^4^ Department of Oncology, the Second Xiangya Hospital, Central South University, Changsha, Hunan, China; ^5^ United Imaging Research Institute of Intelligent Imaging, Radiotherapy (RT) Lab, Beijing, China; ^6^ Central Research Institute, United Imaging Healthcare Company, Shanghai, China; ^7^ Shanghai United Imaging Healthcare Co., Ltd, Radiotherapy (RT) Business Unit, Shanghai, China

**Keywords:** online adaptive radiotherapy (oART), cervical cancer, fan-beam computed tomography (FBCT), image-guided radiotherapy (IGRT), dosimetric distribution, acute toxicity

## Abstract

**Background:**

This study aims to investigate the feasibility of fan-beam computed tomography (FBCT)-guided online adaptive radiotherapy (oART) in radical radiotherapy for cervical cancer.

**Methods:**

Ten patients who underwent radical radiotherapy for cervical cancer were enrolled in this study. All patients received external beam radiation therapy (EBRT) with a prescription dose of 50.4 Gy/28f, and daily oART with FBCT guidance was performed. Dosimetric analysis was conducted on 278 fractions, comparing the adaptive and scheduled plans. The γ passing rate was measured through *in-vivo* dose monitoring during treatment, using a 3%/3mm gamma criterion with an 88% threshold for alerts. The time invested in the oART workflow was recorded at each step. Acute toxicities were classified following the Common Terminology Criteria for Adverse Events (CTCAE) version 5.0.

**Results:**

The adaptive plans demonstrated a dosimetric advantage in target coverage and/or organs at risk (OARs) sparing across all 278 fractions. Compared to the scheduled plan, the adaptive plan showed improved dose received by 95% (D95) of planning target volume (PTV), conformity index (CI), and homogeneity index (HI) (P<0.001). Among the three PTVs, the PTV of uterus (PTV_U) benefited most from dosimetric improvements in the adaptive plan, followed by the PTV of cervix, vagina, and parametrial tissues (PTV_C), while the PTV of lymph node (PTV_N) exhibited the least enhancement. For OARs, the adaptive plan achieved reductions in the dose to the most irradiated 2 cm³ volume (D2cc) for the rectum, bladder, and small intestine (P<0.001). For patients with ovarian conservation, the dose to the 50% volume (D50) and the mean dose of the bilateral ovaries were decreased (P<0.001). The mean γ passing rate across all fractions was 99.24%. The mean duration of the oART workflow was 22.82 ± 3.61 min, with auto-segmentation & review (44.40%) and plan generation & evaluation (22.02%) being the most time-intensive steps. The incidence of Grade 1-2 acute non-hematological toxicity was 60%, with no cases of Grade 3 or higher observed.

**Conclusions:**

The implementation of FBCT-guided oART in radical radiotherapy for cervical cancer was feasible. This approach has shown significant improvements in dose distribution and the potential to provide clinical benefits by reducing acute toxicity.

## Introduction

Cervical cancer is among the most prevalent malignant neoplasms of the female reproductive system, exhibiting a global age-standardized incidence rate of 13.3 per 100,000 and a mortality rate of 7.2 per 100,000 in 2020, notably elevated in low and middle-income countries (1). Radiotherapy, particularly external beam radiotherapy (EBRT) followed by brachytherapy, plays a central role in the definitive management of locally advanced cervical cancer. The evolution of EBRT technology, along with the adoption of image-guided radiotherapy (IGRT), has resulted in better target conformity and reduced doses to organs at risk (OARs) (2, 3). However, dynamic changes in pelvic organ volume and position, such as bladder filling and intestinal gas variations, lead to significant inter-fractional motion of the target volume, making it challenging to deliver precise and effective radiation doses (4–6). Conventionally, inter-fractional motion has been managed by establishing sufficient margins from the clinical target volume (CTV) to the planning target volume (PTV), thereby ensuring adequate coverage. However, this approach increases radiation exposure to surrounding healthy tissues, elevating the risk of treatment-related toxicity. To mitigate these challenges, online adaptive radiotherapy (oART) has emerged as a promising approach to optimize dose delivery by adapting treatment plans to anatomical changes based on daily images (7). Recent advances in artificial intelligence (AI)-driven auto-segmentation and treatment planning have further enhanced the feasibility of oART (8).

Currently, clinical implementation of oART is guided by various imaging modalities, including cone-beam computed tomography (CBCT), magnetic resonance imaging (MRI), and fan-beam computed tomography (FBCT). CBCT is widely utilized for oART; however, its suboptimal image quality and the need for pseudo-CT generation for dose calculations may compromise treatment precision (9, 10). MR-guided radiotherapy has garnered attention for its superior soft-tissue contrast in adaptive workflows, but its widespread adoption is constrained by high cost, restricted availability, and prolonged treatment times (11). FBCT has emerged as a promising imaging modality for oART, offering a balance between high soft-tissue resolution and rapid image acquisition while enabling direct dose calculation on the acquired images. Despite these advantages, clinical research on FBCT-guided oART remains relatively limited.

This study aims to evaluate the implementation of FBCT-guided oART in definitive radiotherapy for cervical cancer. By comparing dosimetric outcomes, analyzing acute treatment-related toxicity and clinical response, as well as evaluating workflow processes, we seek to provide evidence supporting the wider clinical adoption of FBCT-guided adaptive strategies in cervical cancer.

## Materials and methods

### Patients

From May 2023 to August 2023, ten consecutive cervical cancer patients scheduled for undergo radical radiotherapy at Peking Union Medical College Hospital were enrolled in this study. Inclusion criteria included (1) confirmed diagnosis of cervical cancer through imaging and biopsy pathology; (2) indication for radical radiotherapy; (3) life expectancy of more than 6 months; (4) ECOG score of 0-2 and ability to remain lying flat for more than 30 minutes. Patients with a history of pelvic radiotherapy, contraindications to radiotherapy, or need for irradiation of the para-aortic or inguinal lymph node drainage regions were excluded. All patients received EBRT using volumetric modulated arc therapy (VMAT) followed by three-dimensional brachytherapy. All patients received 4-6 cycles of concurrent cisplatin-sensitizing chemotherapy, except one patient who was intolerant to platinum-based drugs. Treatment response was assessed at 1 and 3 months after radiotherapy using MRI and tumor markers. Squamous cell carcinoma antigen (SCC-Ag) was measured for squamous carcinoma, while carcinoembryonic antigen (CEA), cancer antigen 125 (CA125), and cancer antigen 19-9 (CA199) were analyzed for adenocarcinoma. Clinical complete response (cCR) was defined as normal tumor marker levels and the absence of residual tumor or enlarged metastatic lymph nodes on pelvic MRI. Toxicities occurring during radiotherapy and within 3 months after its completion were defined as acute toxicities. These toxicities were graded and recorded according to the Common Terminology Criteria for Adverse Events version 5.0 (CTCAE 5.0).

### CT simulation and reference plan generation

Patients were guided to empty their bladder and bowels 2 hours prior to both the CT simulation and each subsequent fraction. They were then instructed to consume 500 mL of water 1.5 hours before the treatment time and to refrain from urinating until after their session. Following fixation of the patient’s position, a CT scan was conducted that covered an area extending from the superior boundary of the liver to 5 cm beneath the ischial tuberosity, with a scan slice thickness of 5 mm. The target volumes were delineated in accordance with Radiation Therapy Oncology Group consensus guidelines for definitive radiotherapy of cervical cancer (12, 13). The gross target volume of the lymph nodes (GTVnd) was defined as the metastatic lymph nodes visible on the CT/MRI/PET images. The CTV consisted of three parts, including the CTV of the lymph nodes (CTV_N), the CTV of the uterus (CTV_U), and the CTV of the cervix, vagina, and parametrial tissues (CTV_C). CTV_N encompassed the common iliac, internal iliac, external iliac, obturator, and presacral lymph node drainage regions. CTV_U was expanded isotropically by 10 mm to form the PTV of the uterus (PTV_U), while CTV_C and CTV_N were each expanded isotropically by 5 mm to generate the PTV of the cervix, vagina, and parametrial tissues (PTV_C) and the PTV of the lymph node (PTV_N), respectively. These three PTVs were then combined to form the overall PTV. GTVnd expanded a 5mm margin to create the PTV of the metastatic lymph nodes (PTVnd). The prescribed dose for the three PTVs was 50.4 Gy in 28 fractions, with a simultaneous boost of 60.2 Gy in 28 fractions to PTVnd. A dual-arc VMAT reference plan was created based on the clinical goal sheet (**Supplementary Table S1**) for treatment implementation, using a 2.5 mm dose grid spacing. All treatment plans in this study were generated using the United Treatment Planning System (uTPS, Shanghai United Imaging Healthcare Co., Ltd, Shanghai, China). The reference plan was manually designed using the Stochastic Platform Optimization (SPO) algorithm (14).

### The workflow of daily oART

The oART for the definitive treatment of cervical cancer was performed using the uRT-linac 506c (Shanghai United Imaging Healthcare Co., Ltd, Shanghai, China), which is a 6-megavolt (MV) C-arm linear accelerator integrated with a diagnostic-quality kV FBCT. The X-ray linac and FBCT are co-axially equipped on the same bed, simultaneously realizing CT simulation, daily FBCT guidance, and beam delivery on the same platform. Detailed information about this machine has been previously reported (15).

**Figure 1:**
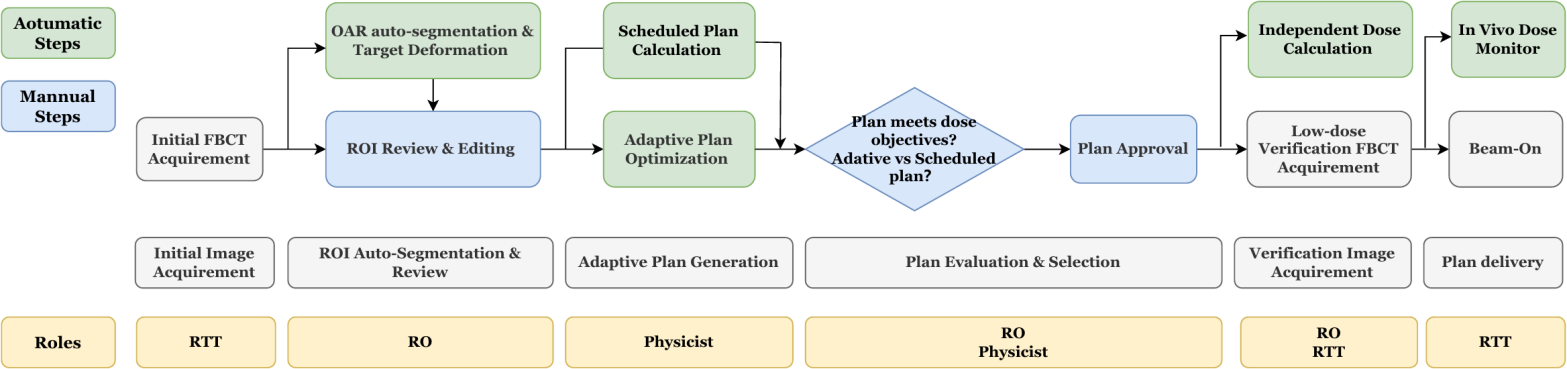
The workflow of FBCT-guided daily online adaptive radiotherapy. RO, radiation oncologist; RTT, radiotherapy technologist.

**Figure 2:**
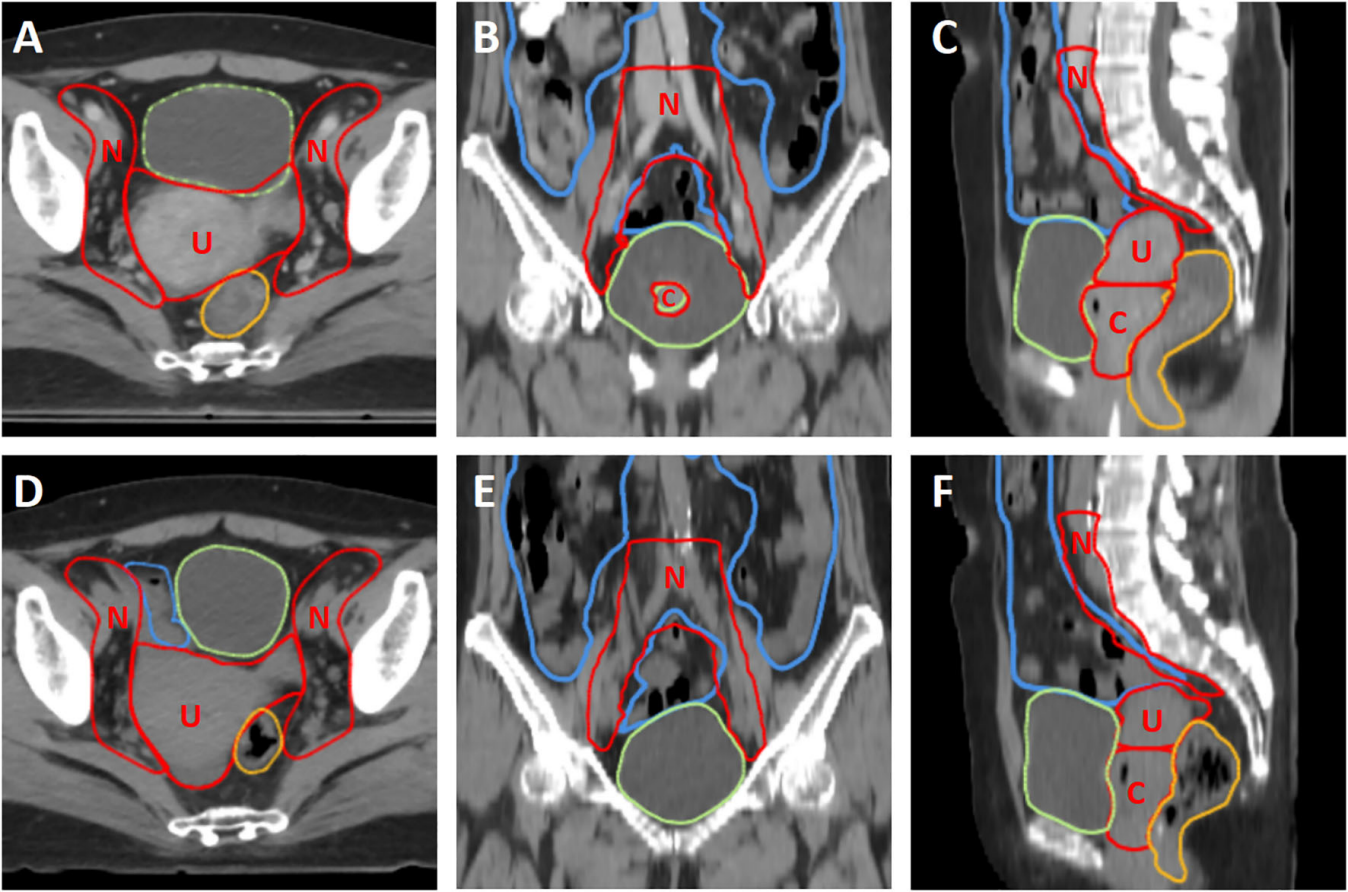
The comparison of simulation CT and daily kV FBCT of a representative patient. **(A–C)** The first row shows the simulation CT in axial, coronal, and sagittal planes, respectively. **(D–F)** The second row shows the daily kV FBCT in axial, coronal, and sagittal planes, respectively. The window level and window width are set to 40 and 400, respectively. Red contours represent the three CTVs (CTV_U, CTV_C, and CTV_N, labeled as U, C, and N), while green, yellow, and blue contours represent the bladder, rectum, and small intestine, respectively.

As shown in **Figure 1**, the oART workflow mainly consisted of six steps: initial image acquisition, region of interest (ROI) auto-segmentation and review, adaptive plan generation, plan evaluation and selection, verification image acquisition, and treatment delivery. The oART workflow started with the initial FBCT acquisition. The FBCT scan covered an area extending more than 5 cm beyond the superior and inferior boundaries of the PTV volume with a scan slice thickness of 5 mm. The contours of the three CTVs were generated through deformable registration. The contours of all OARs were delineated using an AI-based auto-segmentation algorithm applied to daily FBCT images to account for inter-fractional anatomical changes. The auto-segmentation algorithm is based on a lightweight deep learning framework for radiotherapy treatment planning (RTP-Net). The algorithm supports whole-body OARs auto-segmentation with a level of accuracy comparable to, if not superior to, that of manual delineation, as evidenced by a mean Dice Similarity Coefficient (DSC) of 0.95. Furthermore, the algorithm facilitates real-time segmentation, with most tasks completed in under two seconds (16). Subsequently, the radiation oncologist reviewed and edited the ROI contours. **Figure 2** shows the daily FBCT images of a representative patient. Compared with the simulation CT, the image quality remained consistent, whereas there were significant differences in the anatomical position of the CTV, which were attributed to variations in bladder filling and intestinal movement.

Once the contours review was completed, the adaptive plan was automatically created and optimized with the dose directly calculated on the daily kV FBCT images. This process utilized a fully automated algorithm that took the dose distribution of the reference plan and the clinical goal sheet as inputs. First, the clinical goal sheet was used to establish optimization constraints for the adaptive plan. Second, the dose-volume histograms (DVHs) of OARs were predicted by extracting the dose falloff features from the reference plan, which were used to update the optimization objectives. Third, target-related dosimetric parameters from the reference plan, including the conformity index (CI), dose at 2% and 98% volumes, and minimum and maximum doses, were extracted to guide the optimization process. Furthermore, the algorithm implemented various optimization strategies, including OAR dose reduction, dose conformity optimization, and hotspot removal, to ensure that the plan quality met the clinical requirements. Additionally, the scheduled plan was generated by mapping the reference plan to the daily kV FBCT images and recalculating the dose distribution.

Next, the workflow automatically proceeded to the plan evaluation and selection module. The radiation oncologist compares the clinical goals, dose distribution, and DVH of the adaptive and scheduled plans for evaluation and makes the final decision on plan selection manually. After plan approval, a validation FBCT was acquired using a low-dose protocol (one-third of the regular dose) to monitor intra-fractional changes. Meanwhile, the system automatically exported the selected plan to independent dose calculation software for online patient-specific quality assurance, which was conducted using a Monte Carlo-based independent dose calculation method tool uAssureTx.

During plan delivery, an electronic portal imaging device (EPID) was used to monitor the *in-vivo* doses of radiation fields. The transmission image was calculated using the Monte Carlo algorithm, taking into account the phase space of photons and electrons, detector response, and lateral scatter. The measured images were corrected before gamma comparison. To elaborate, geometry correction, dead pixel correction, dark current correction, and detector response correction were performed (17). Each patient underwent EPID *in vivo* monitoring for every fraction. A 3%/3mm gamma comparison between calculated transmission image and measured image is performed for every 30° of gantry rotation in real-time during delivery, and the passing rates are displayed on the treatment console. Based on previous studies and internal organ motion in real patient cases, we set the threshold of gamma passing rate to 88% (17, 18). If it falls below this threshold, the system alerts the therapist to assess treatment termination. The average passing rate overall delivered arc sections was calculated and reported for that fraction.

### Statistical analysis

Mean and standard deviation (SD) were used for the analysis to describe continuous variables. Statistical tests with P<0.05 (two-sided) were considered significant. The Kolmogorov-Smirnov method was used to test the normality of the data. The dose difference between scheduled and adaptive plans was compared using the Wilcoxon signed-rank test for data with a non-normal distribution. The dose metrics were based on the addition of average value in the metrics across each delivered fraction (19). Boxplots showing the median value and interquartile range (IQR) were created with outliers outside 1.5 × IQR.

All statistical analyses were performed using SPSS version 26 (IBM Corp., Armonk, New York, USA) and R version 4.3.0 (R Core Team, R Foundation for Statistical Computing, Vienna, Austria), and GraphPad Prism 9 (GraphPad Software, La Jolla, California, USA).

## Results

### Patients characteristics

The clinical characteristics of the patients are summarized in **Table 1**. The average age of the enrolled patients was 54.7 ± 15.5 years. The FIGO stages ranged from IB3 to IIIC1, with squamous cell carcinoma being the predominant histological type, identified in 9 of 10 patients. Patients 3 and 10 underwent ovarian transposition surgery followed by ovarian-sparing radiotherapy. While all patients were scheduled for standard concurrent chemoradiotherapy, Patient 7 received immunotherapy with tislelizumab as a substitute for platinum-based chemotherapy due to intolerance. A total of 278 fractions were administered using oART technology. However, due to equipment maintenance or the absence of a radiation oncologist to delineate the target area, the remaining two treatments were conducted using the reference plan after confirming that the targets were not missed with FBCT.

**Table 1:** Clinical characteristics of 10 cervical cancer patients treated with the oART technique.

No.	Age	FIGO 2018 staging	Histopathology	Pelvic node boost	Ovarian Protection	Brachytherapy	Concurrent chemotherapy	Weight change (KG)	Acute toxicity grade*				Clinical Outcomes
									Rectum	UGI	Urinary	Hematologic	
1	55	IIIC1	Adeno	Yes	No	30Gy/5f	CDDP*6	+0.4	0	0	0	2	PR
2	58	IIA1	SCC	No	No	30Gy/5f	CDDP*5	-1.0	0	1	0	2	CR
3	30	IIB	SCC	No	Yes	30Gy/5f	CDDP*4	0	1	2	0	2	CR
4	57	IIIC1	SCC	Yes	No	30Gy/5f	CDDP*4	-5.3	0	2	0	3	CR
5	73	IIB	SCC	No	No	30Gy/5f	CDDP*4	-2.4	0	1	0	3	CR
6	53	IIIC1	SCC	Yes	No	30Gy/5f	CDDP*5	+0.1	0	0	0	3	CR
7	81	IIB	SCC	No	No	30Gy/5f	No	+1.0	0	0	0	3	CR
8	44	IB3	SCC	No	No	36Gy/6f	CDDP*5	+1.5	0	0	0	1	CR
9	60	IIB	SCC	No	No	30Gy/5f	CDDP*5	+1.6	0	1	0	2	CR
10	36	IIB	SCC	No	Yes	30Gy/5f	CDDP*5	-1.5	2	1	0	1	CR

FIGO, International Federation of Gynecology and Obstetrics; Adeno, adenocarcinoma; SCC, squamous cell carcinoma; CDDP, cis-diamminedichloroplatinum; UGI, upper gastrointestinal tract; CR, complete remission; PR, partial remission.

*Acute toxicity was graded by the CTCAE 5.0 version.

### Duration for the workflow of oART

**Figure 3:**
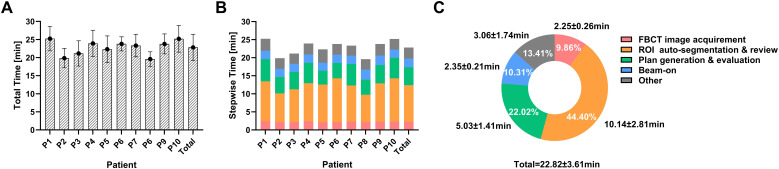
The duration for the workflow of oART. **(A)** Total duration of oART in each patient and overall. **(B, C)** Allocation of time within the various segments of oART in each patient and overall.

The mean ± SD duration for the oART workflow was 22.82 ± 3.61min (**Figures 3A–C**), ranging from 15.58 to 38.45 minutes. The interquartile ranged from 20.39 to 24.63 minutes. The total time was recorded from the initial image acquisition to the completion of treatment delivery. The breakdown of the total time spent on the oART process is depicted in **Figure 3C**, including FBCT image acquisition and registration (2.25 ± 0.26min, 9.86%), ROI auto-segmentation & review (10.14 ± 2.81min, 44.40%), plan generation & evaluation (5.03 ± 1.41min, 22.02%), and beam-on (2.35 ± 0.21min, 10.31%). The remaining time (3.06 ± 1.74min, 13.41%) was allocated to transitional activities, including data transfer, equipment gantry adjustments, etc. The most time-intensive steps in the entire process were ROI auto-segmentation & review (44.40%) and plan generation & evaluation (22.02%), which exhibited the most significant variability among different patients and different treatment fractions.

### Comparison of dosimetric results between adaptive and scheduled plans

**Figure 4:**
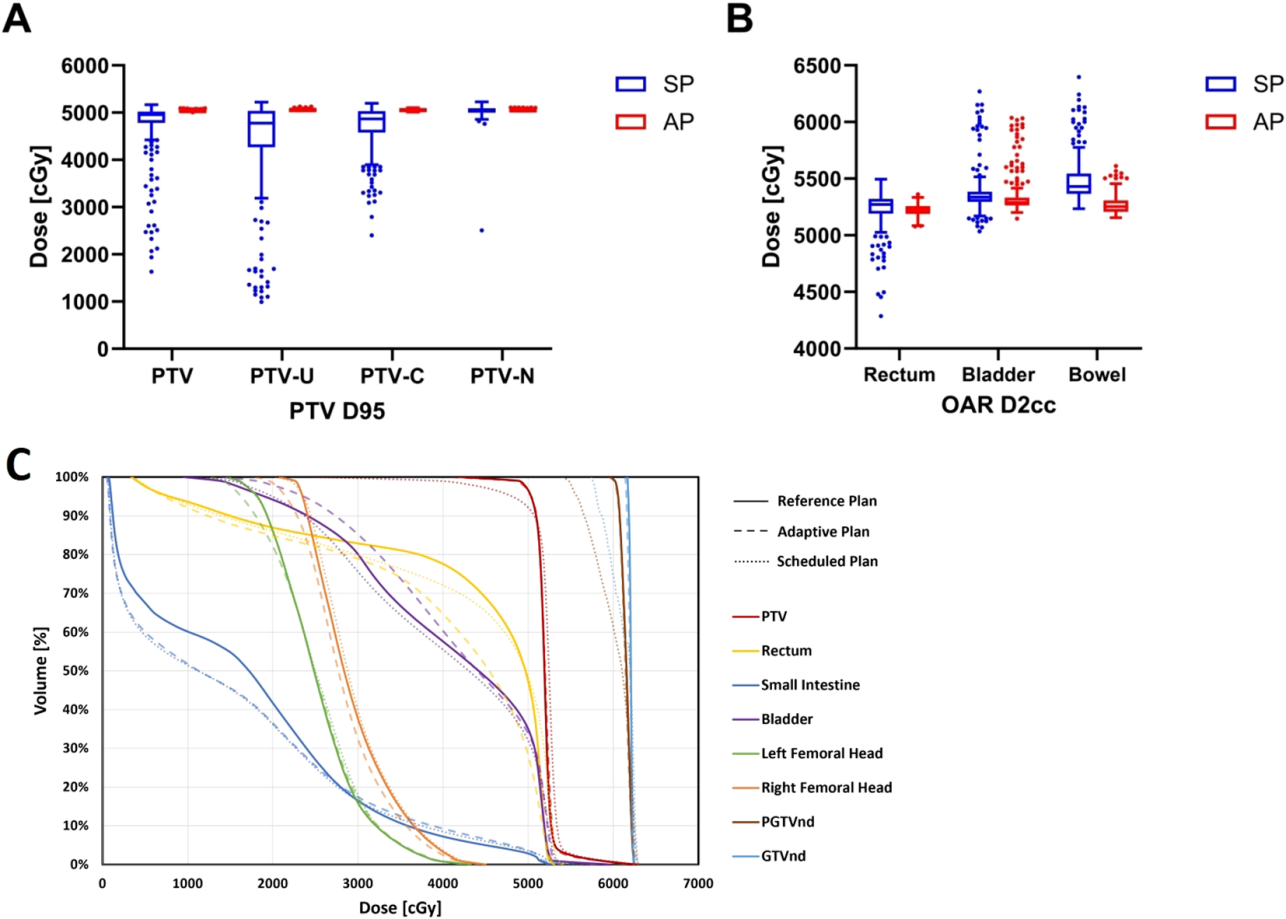
The differences in dosimetric parameters of targets and OARs between adaptive and scheduled plans: **(A)** PTV D95 **(B)** OARs D2cc **(C)** Dose-volume histograms of the three plans for a representative patient.

**Table 2** and **Figures 4A, B** illustrate the dosimetric differences of the targets and OARs between the adaptive and scheduled plans.

**Table 2:** The difference in dosimetric parameters between adaptive plans and scheduled plans.

	Dosimetric Parameter (units)	Adaptive plans Mean ± SD	Scheduled plans Mean ± SD	Difference Mean ± SD	P-value
PTV	D95 (Gy)	50.43 ± 0.13	47.49 ± 6.01	2.94 ± 6.01	<0.001*
	CI (%)	0.90 ± 0.03	0.82 ± 0.06	0.08 ± 0.07	<0.001*
	HI (%)	0.13 ± 0.08	0.26 ± 0.18	-0.13 ± 0.19	<0.001*
PTV_U	D95 (Gy)	50.58 ± 0.18	44.11 ± 9.77	6.47 ± 9.79	<0.001*
PTV_C	D95 (Gy)	50.55 ± 0.17	47.14 ± 4.73	3.42 ± 4.72	<0.001*
PTV_N	D95 (Gy)	50.54 ± 0.16	50.39 ± 1.67	0.15 ± 1.70	0.527
Rectum	D2cc (Gy)	52.22 ± 0.49	52.32 ± 1.62	-0.10 ± 1.68	<0.001*
	Dmean (Gy)	35.17 ± 3.70	35.29 ± 5.99	-0.12 ± 5.71	0.049*
	D50 (Gy)	38.36 ± 5.03	39.26 ± 9.51	-0.89 ± 9.81	<0.001*
Bladder	D2cc (Gy)	53.39 ± 1.62	53.67 ± 1.79	-0.28 ± 1.15	<0.001*
	Dmean (Gy)	36.05 ± 4.01	35.38 ± 7.15	0.68 ± 4.76	0.900
	D50 (Gy)	36.31 ± 6.16	36.34 ± 4.74	-0.03 ± 7.44	0.240
Small Intestine	D2cc (Gy)	52.70 ± 0.80	54.86 ± 1.91	-2.17 ± 1.57	<0.001*
	Dmean (Gy)	17.59 ± 3.34	17.57 ± 3.52	0.02 ± 0.73	0.896
	D50 (Gy)	11.91 ± 6.05	11.91 ± 5.48	0.00 ± 1.71	0.973
Bone Marrow	D90 (Gy)	14.15 ± 1.63	15.04 ± 1.60	-0.89 ± 0.78	<0.001*
Left Femoral Head	D5 (Gy)	31.50 ± 3.11	31.16 ± 3.14	0.33 ± 2.32	0.286
Right Femoral Head	D5 (Gy)	31.28 ± 3.48	31.47 ± 3.58	0.19 ± 1.92	0.897
Spinal Cord	D0.1cc (Gy)	19.25 ± 4.28	22.74 ± 5.89	-3.49 ± 3.10	<0.001*
Left Ovary	Dmean (Gy)	4.03 ± 0.36	7.01 ± 0.88	-2.97 ± 0.91	<0.001*
	D50 (Gy)	3.93 ± 0.31	6.51 ± 0.60	-2.58 ± 0.65	<0.001*
Right Ovary	Dmean (Gy)	3.96 ± 0.24	6.96 ± 2.01	-3.00 ± 1.95	<0.001*
	D50 (Gy)	3.72 ± 0.24	6.46 ± 1.99	-2.73 ± 1.93	<0.001*

*highlights significant p-values.

SD-standard deviation; PTV-planning target volume; CTV-clinical target volume; D95, D50, D5, D2cc, D0.1cc-the dose received by 95%, 50%, 5%, 2cc, 0.1cc of volumes; CI-conformity index = (Vt,ref/Vt)·(Vt,ref/Vref), Vt,ref is the target volume covered by the reference isodose line, Vt is the target volume, Vref is the total volume covered by the reference isodose line; HI-homogeneity index = (D2-D98)/D50.

For target dose distribution, the adaptive plan showed that the mean dose received by the 95% volume (D95) of PTV was 50.43 ± 0.13 Gy, which met the prescribed dose, in contrast to the scheduled plan’s 47.49 ± 6.01 Gy (P<0.001). The adaptive plan offered significant improvements in the conformity index (CI) (0.90 vs. 0.82) and heterogeneity index (HI) (0.13 vs. 0.26) of the PTV compared to the scheduled plan (P<0.001). The adaptive plan also showed an increase in D95 of 6.74 Gy and 3.42 Gy for PTV_U and PTV_C, respectively (P<0.001), although the PTV_N D95 increased by only 0.15 Gy and was not statistically significant.

Regarding the dosimetric comparison for OARs, the adaptive plan yielded reductions in the dose to the most irradiated 2 cm3 volume (D2cc), the mean dose (Dmean), and the dose to the 50% of volume (D50) for the rectum by 0.10Gy, 0.12Gy, and 0.89Gy, respectively (P<0.05). For the bladder and small intestine, the adaptive plan showed a decrease in D2cc by 0.28Gy and 2.17Gy, respectively (P<0.001), although no significant dosimetric benefits were seen in the Dmean and D50 for these OARs. Furthermore, the dose to the 90% volume (D90) of bone marrow and the dose to the most irradiated 0.1 cm3 volume (D0.1cc) of spinal cord in the adaptive plan were significantly improved compared to the scheduled plan (P<0.001). However, no dosimetric benefits for the bilateral femoral heads were observed in the adaptive plans. Notably, for the two patients undergoing ovarian-sparing radiotherapy, the adaptive plan halved the D50 and Dmean for bilateral ovaries to approximately 3-4 Gy (P<0.001).

According to **Figure 4C**, the DVH illustrates the dose disparities across the three treatment options. In terms of target dose distribution for PTV and PTVnd, the adaptive plan showed comparable results to the reference plan and notably superior results than the scheduled plan. The adaptive plan demonstrated an obvious improvement over the scheduled plan in terms of rectal dose distribution.

Analysis of target coverage alongside a priority 1 OAR constraint revealed a dosimetric benefit for the adaptive plans in all 278 fractions. The adaptive plan demonstrated improvement in 222 fractions by providing enhanced target coverage and reduced small intestine D2cc exposure. In 2 fractions, it achieved superior target coverage with comparable small bowel D2cc to the scheduled plan. Meanwhile, in the remaining 54 fractions, the adaptive plan showed better performance in terms of protecting the small bowel D2cc, while both plans delivered a prescribed dose of at least 50.40 Gy to the PTV D95.

### Independent dose verification and *in vivo* dose monitor

Independent dose verification revealed that out of 278 evaluations, the 3%/3mm γ passing rate was an exceptional 99.55 ± 0.29%. Notably, the lowest recorded γ passing rate was a remarkable 98.40%, comfortably surpassing the commonly accepted threshold for clinical requirements.

**Figure 5:**
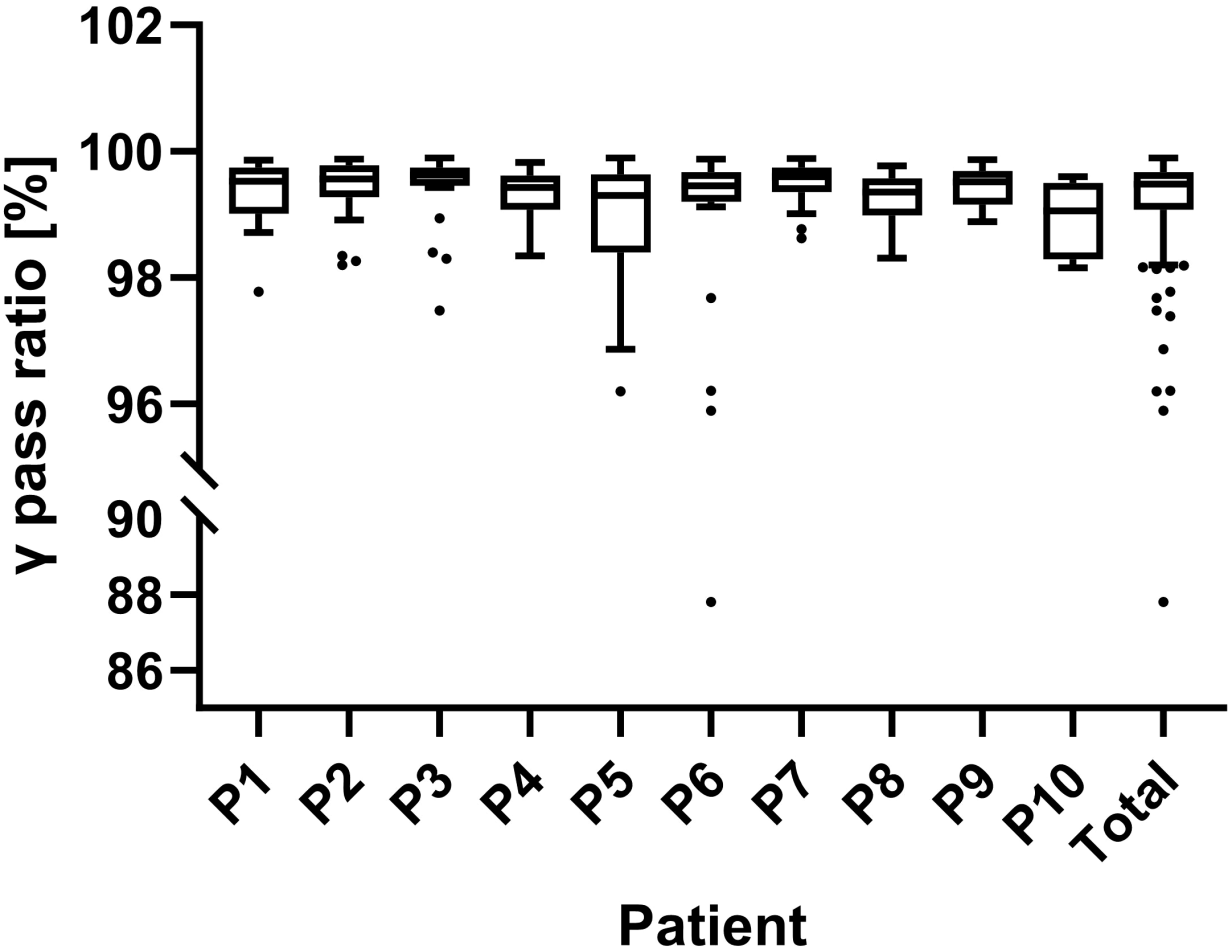
Results of *in-vivo* dose monitoring using an electronic portal imaging device (EPID). The γ passing rates for each patient and overall are shown. A 3%/3mm gamma criterion was used for real-time verification, with passing rates assessed every 30°of gantry rotation and an 88% threshold for alerts.

During plan delivery, EPID was used to monitor the *in-vivo* doses of radiation fields. **Figure 5** illustrates that the implementation of two-dimensional *in vivo* dose monitoring during treatment administration yielded an average γ passing rate of 99.24 ± 1.35% across 278 fractions. All patients demonstrated a mean γ passing rate exceeding 99%, except for Patients 5, 6, and 10. Patients 5 and 10 had lower average gamma passing rates due to significant intestinal gas variations in some fractions. Patient 6 had a low gamma passing rate in the first four fractions because of insufficient CT scan range, but this issue was corrected in subsequent fractions.

### Clinical outcomes and acute toxicities

The clinical outcomes and acute toxicities of the patients are presented in **Table 1**. The median follow-up time for all patients was 3.0 months (range: 3-4 months). At 3 months after radiotherapy, 90% of the patients achieved cCR. However, one patient showed persistent abnormal MRI signals, indicating residual tumor after treatment. The pathological type of this patient was gastric adenocarcinoma, which is known to be insensitive to radiotherapy.

In terms of acute toxicity, all patients experienced varying degrees of hematopoietic toxicity (Grade 1-3). The incidence of Grade 1-2 acute non-hematological toxicity in all patients was 60%, whereas no instances of Grade 3 or above acute non-hematological toxicity were observed. Upper gastrointestinal toxicity was common acute toxicity, with 2 cases of Grade 2 and 4 cases of Grade 1. Rectal toxicity mainly manifested as diarrhea, with 1 case of Grade 2 and 1 case of Grade 1. No patient experienced urinary toxicity. All patients fully recovered from acute toxicity at 3 months after the end of radiotherapy.

During radiotherapy, the patients’ weight fluctuated slightly, with a mean weight change of -0.56 ± 2.11 Kg. Patient 4, however, experienced a significant weight loss of 5.4 Kg, which was attributed to poor appetite caused by Grade 2 upper gastrointestinal toxicity.

## Discussion

Cervical cancer, due to the fact that it is significantly affected by physiological changes in the pelvic organs, is a typical disease that is suitable and necessary for oART (20). In this study, different from previous studies, diagnostic-quality FBCT was applied for oART image guidance. A comprehensive analysis was conducted on the workflow, dosimetry, and clinical outcomes associated with FBCT-guided daily oART in cervical cancer.

High-quality image guidance is a key foundation for implementing oART. The uRT-Linac 506c, utilized in this study, represents a CT-linac integration that combines kV FBCT with a 6-MV X-ray linac in a coaxial arrangement. This kV FBCT has been shown to achieve a spatial resolution of ≥15 line pairs per millimeter (lp/mm) and a low-contrast detectability of 2 mm (12). The acquired image sequence of FBCT shares a CT value-relative electron density conversion curve with the simulation CT, making it used directly for adaptive plan generation (15). While offering high spatial resolution, the imaging dose of FBCT is comparable to that of CBCT, alleviating concerns about increased patient radiation exposure with daily CT guidance. Currently, low-dose FBCT has already been adopted in clinics to reduce dosages further. Wei Gong et al. demonstrated that low-dose FBCT can achieve peripheral doses as low as 1.85mGy at the scanning field, highlighting its potential as a promising direction for future optimization (21, 22). In summary, FBCT offers a combination of high spatial resolution, direct usability for dose calculation and plan design, acceptable imaging radiation dose, and seamless integration with linear accelerators in a coaxial setup, making it a promising image guidance modality for oART.

The oART is a labor-intensive and time-consuming procedure; thus, minimizing the duration of the process is imperative for its broader adoption in clinical practice. In this study, the average duration of the oART workflow was 22.82 ± 3.61min, similar to the reported average of 21-29 minutes in previous literature on daily oART for cervical cancer (20, 23, 24). In terms of the time composition, the ROI auto-segmentation & review was the most time-consuming step (44.40%), followed by the plan generation & evaluation (22.02%), which was also highlighted in the study by Shelley et al. (24). Considering the major speed-limiting steps in oART, further developments of AI-based deformable registration algorithms and dose optimization algorithms are crucial for enhancing oART efficiency (25). In addition, identifying the appropriate treatment fractions to trigger oART is an important approach to saving time and labor and maximizing clinical efficiency. Previous research has proposed various potential triggers, including weight loss, tumor shrinkage, changes in body shape, and significant deviations from dosimetric objectives for target coverage and OAR dose constraints, though the optimal timing for these triggers is still under investigation (26–28). Furthermore, there is growing interest in using machine learning and deep learning techniques to identify image features and develop predictive models for triggering oART, with relevant research conducted in pancreatic (29, 30) and lung cancers (31). The high-quality images provided by FBCT offer promising prospects for developing similar models for cervical cancer.

oART has obvious dosimetric advantages in cervical cancer radiotherapy and can deliver dose accurately. In this study, the adaptive plan achieved a mean PTV D95 exceeding the prescribed dose, ensuring superior conformity and homogeneity. In contrast, scheduled plans failed to meet the prescribed dose for mean PTV D95, potentially resulting in underdosed regions, or “cold spots,” within the target volume, which could compromise tumor control. Notably, **Figure 4A** clearly illustrates the substantial variability in target coverage across different fractions in the scheduled plan, with numerous outliers in PTV D95. In contrast, the adaptive plan ensured that PTV D95 for nearly all fractions remained consistently close to the prescribed dose, demonstrating its robustness in ensuring treatment precision. Regarding normal tissue sparing, oART reduced radiation exposure to critical organs, including the small intestine, rectum, and bladder, though with modest absolute reductions. Notably, the ovarian dose was reduced by 50%, which could have significant clinical implications for ovarian function preservation. These findings align with previous studies that have demonstrated the dosimetric benefits of adaptive plans over scheduled plans (23, 24). Furthermore, EPID-based *in vivo* dose monitoring confirmed a mean γ passing rate above 98.5% for all patients, verifying the accuracy of dose delivery in FBCT-guided oART and further supporting its clinical feasibility.

Differential dosimetric advantages were observed within various sub-volumes of the target volumes. The PTV_U derived the most significant benefit from the adaptive plan, followed by PTV_C, whereas the PTV_N experienced the least improvement. This variation may be attributable to the uterus being more susceptible to movements from the adjacent rectum and bladder, while the position of lymph nodes remains relatively static. It is thus sensible to tailor the margins from CTV to PTV for different parts of the target volume, considering the distinct motion characteristics of various anatomical structures, to ensure adequate target coverage while minimizing exposure to surrounding healthy tissues. Based on our previous participation in an international multicenter study (32) and literature reports (33), we typically use a 15mm margin for CTV_U and CTV_C, and a 6-8mm margin for CTV_N in IGRT for cervical cancer in our hospital. There are no uniform conclusions regarding the PTV margins for daily oART of cervical cancer. However, previous studies on intra-fractional motion during radiotherapy for cervical cancer provide valuable insights that can guide margin selection. Guangyu Wang et al. investigated daily oART for postoperative cervical and endometrial cancer patients, finding that a uniform 5mm expansion ensured full coverage of the nodal CTV in 100% of fractions in the validation cohort. They further suggested that the margin could be reduced to 4 mm if >95% nodal CTV coverage was maintained (34). Extensive research on intra-fractional motion during adaptive radiotherapy for cervical cancer has consistently demonstrated that a 5mm margin is sufficient to achieve 95-98% CTV coverage (35, 36). However, the uterus, particularly at the fundus, exhibits substantial intra-fractional motion and is more susceptible to bladder filling variations (33). Given the high mobility of the uterine region, a CTV to PTV margin of 1cm has been recommended to ensure adequate coverage for the uterine fundus (37). In light of these findings, our study adopted a region-specific margin strategy to optimize target coverage while minimizing unnecessary dose exposure. A 5mm margin was applied to generate PTV_C and PTV_N, whereas a larger 10mm margin was used for PTV_U to compensate for the pronounced motion of the uterus.

The clinical benefits of the application of oART in cervical cancer in terms of reduction of treatment toxicity is currently unclear, and the available data on toxicity evaluation from previous studies are minimal. The incidence of Grade 1 and Grade 2 acute non-hematological toxicities (urinary, rectal, and upper gastrointestinal) was 60%, with no occurrences of Grade 3 or higher acute non-hematological toxicities. Comparing previous data from cervical cancer patients receiving IMRT at our medical center, Grade 1 and Grade 2 acute non-hematological toxicity occurred in 86.1% of patients (38), which is significantly higher than the incidence in patients receiving oART. Similar results were found in comparison with studies in other medical centers (39, 40). This preliminary evidence suggests the potential translation of dosimetric advantages of oART into clinical benefits, although robust support from large-sample clinical research data is still required.

To the best of our knowledge, this study represents the first prospective research to implement FBCT-guided daily oART for radical radiotherapy of cervical cancer. However, several limitations must be acknowledged. First, the study included only 10 cervical cancer patients with a short follow-up period, which limits the representativeness and statistical power of the findings. Consequently, we can only preliminarily conclude that FBCT-guided daily oART has the potential to reduce acute treatment-related toxicities in cervical cancer patients. Future large-scale, multicenter, randomized controlled trials are needed to further validate the clinical benefits of this technology. Second, patients with cervical cancer requiring radiotherapy in the para-aortic or inguinal lymphatic drainage areas were excluded due to the additional time required for delineation in these regions. In future studies, a more diverse patient population, with varying individual and disease characteristics, should be included to explore whether FBCT-guided oART can offer efficient workflows and dose advantages in a broader range of patients. Third, this study did not conduct an in-depth analysis of anatomical changes in the target volume and OARs between pre- and post-treatment FBCT images. Consequently, there is a lack of investigation into the magnitude of intra-fractional motion and its influencing factors. As a result, the present study is unable to provide more specific recommendations regarding the optimal CTV-PTV margins in clinical applications of oART. Fourth, oART is both labor-intensive and time-consuming, and the default daily oART strategy in this study imposes a significant burden on the radiotherapy team. In future studies, identifying the appropriate treatment fractions for triggering oART is essential for conserving time and labor resources, which is critical for facilitating its broader clinical adoption.

## Conclusion

The implementation of FBCT-guided oART in radical radiotherapy for cervical cancer was feasible. This approach has shown significant improvement in dose distribution and reliable dose delivery accuracy. Furthermore, it has been found to have an acceptable workflow time and the potential to provide clinical benefits by reducing acute toxicity. Further multicenter studies are essential to corroborate its clinical benefits. The popularization of this technology hinges on the refinement of the oART process and the development of a robust triggering model.

## Data Availability

The original contributions presented in the study are included in the article/supplementary material. Further inquiries can be directed to the corresponding authors.
