# Supplementary material for: Evaluating the Implementation of fan-beam CT-guided online adaptive re-planning in definitive cervical cancer radiotherapy
Source: Front Oncol. 2025 Mar 21;15:1509619. doi: 10.3389/fonc.2025.1509619 (PMC11968674; doi:10.3389/fonc.2025.1509619)
Supplement: Supplementary file 1 [file Table1.docx]

**Table S1 Dose objectives and priorities of regions of interest for optimization.**

| **ROI** | **Clinical dose objective** | **Priority** |
| --- | --- | --- |
| PTV_C | Prescription dose=50.4 Gy | 1 |
| PTV_C | maximum dose≤53.5 Gy | 1 |
| PTV_C | 95% volume to receive≥100%  of prescription (50.4 Gy) | 1 |
| CTV_C | Prescription dose=50.4 Gy | 1 |
| CTV_C | minimum dose≥51 Gy | 1 |
| PTV_N | Prescription dose=50.4 Gy | 1 |
| PTV_N | maximum dose≤53.5 Gy | 1 |
| PTV_N | 95% volume to receive≥100%  of prescription (50.4 Gy) | 1 |
| CTV_N | Prescription dose=50.4 Gy | 1 |
| CTV_N | minimum dose≥51.0 Gy | 1 |
| PTV_U | Prescription dose=50.4 Gy | 1 |
| PTV_U | maximum dose≤53.5 Gy | 1 |
| PTV_U | 95% volume to receive≥100%  of prescription (50.4 Gy) | 1 |
| CTV_U | Prescription dose=50.4 Gy | 1 |
| CTV_U | minimum dose≥51 Gy | 1 |
| Bladder | mean dose≤32 Gy | 2 |
| Bladder | ＜50% volume to receive≥35 Gy | 2 |
| Rectum | mean dose≤35 Gy | 2 |
| Rectum | ＜50% volume to receive≥38 Gy | 2 |
| Small Intestine | mean dose≤35 Gy | 2 |
| Small Intestine | ＜50% volume to receive≥20 Gy | 2 |
| Small Intestine | ＜2cc volume to receive≥52 Gy | 1 |
| Spinal Cord | ＜0.1cc volume to receive≥30 Gy | 2 |
| Bone Marrow | mean dose≤28 Gy | 3 |
| Bone Marrow | ＜90% volume to receive≥15 Gy | 3 |
| Femur_Head_R | mean dose≤30 Gy | 3 |
| Femur_Head_R | ＜5% volume to receive≥30 Gy | 3 |
| Femur_Head_L | mean dose≤30 Gy | 3 |
| Femur_Head_L | ＜5% volume to receive≥30 Gy | 3 |
| Ovary_R* | mean dose≤3.8 Gy | 1 |
| Ovary_R* | ＜50% volume to receive≥4 Gy | 1 |
| Ovary_L* | mean dose≤3.8 Gy | 1 |
| Ovary_L* | ＜50% volume to receive≥4 Gy | 1 |

* The dose constraints applied when employing the ovarian protection regimen in young patients.
